# Perioperative Ischemic Stroke in Unruptured Intracranial Aneurysm Surgical or Endovascular Therapy

**DOI:** 10.7759/cureus.7645

**Published:** 2020-04-12

**Authors:** Hind A Beydoun, May Beydoun, Alan Zonderman, Shaker M Eid

**Affiliations:** 1 Research Programs, Fort Belvoir Community Hospital, Fort Belvoir, USA; 2 Intramural Research Program, National Institute on Aging, Baltimore, USA; 3 Hospital Medicine, Johns Hopkins University School of Medicine, Baltimore, USA

**Keywords:** aneurysm, endovascular, coiling, clipping, ischemic stroke, surgical

## Abstract

Background: Ischemic stroke among hospitalized patients who underwent surgical or endovascular therapies for unruptured intracranial aneurysms (IAs) has not been previously examined in nationally representative samples.

Objectives: The purpose of this study is to compare the occurrence and in-hospital outcomes (nonroutine discharge, length of stay) of perioperative ischemic stroke among hospitalized patients diagnosed with unruptured IA across treatment selection [surgical clipping, endovascular coiling, stent- or balloon-assisted coiling (SAC or BAC), combined clipping and coiling].

Methods: A cross-sectional study was conducted using 23,053 hospital discharge records from the 2002-2012 Nationwide Inpatient Sample (NIS). Rates, β coefficients, and odds ratios (ORs) with their 95% confidence intervals (CIs) were calculated accounting for survey design complexity, patient- and hospital-level confounders.

Results: Ischemic stroke rate was 6.9% [surgical clipping (4.3%), endovascular coiling (8.1%), BAC or SAC (1.9%), and combined techniques (4.2%)]. Multivariable logistic regression models suggested that compared to patients undergoing surgical clipping alone, those undergoing SAC or BAC were less likely to be diagnosed with ischemic stroke (adjusted OR=0.34, 95% CI: 0.14, 0.85). Compared to clipping, endovascular techniques resulted in fewer nonroutine dispositions and shorter hospitalizations, whereas combined techniques resulted in longer hospitalizations. Differences in hospitalization outcomes between treatment types were only affected by ischemic stroke when comparing coiling to clipping.

Conclusions: Perioperative ischemic stroke rate among patients with unruptured IA may be less among those undergoing SAC or BAC as compared to those undergoing surgical clipping alone. Improved in-hospital outcomes among coiling versus clipping recipients may depend on ischemic stroke diagnosis.

## Introduction

Stroke is a leading cause of disability resulting from aphasia, hemiparesis, depression, rehabilitation, long-term care, and dependence on individuals and assisted devices [1-2], with considerable healthcare expenditures linked to this chronic condition [1, 3]. While ischemic stroke represents 80%-85% of all strokes, intracerebral hemorrhage and subarachnoid hemorrhage (SAH) are two distinctive forms of hemorrhagic stroke, representing 15%-20% of strokes [3-4]. Nearly 5%-6% of strokes consist of SAH with an incidence rate of nine per 100,000 and a case fatality rate of 50%-60% if inadequately treated [3-5]. A patient’s prognosis after SAH depends on the occurrence of complications such as vasospasm, hydrocephalus, and delayed ischemic deficit as well as clinical condition at the time of presentation and other factors [5].

Intracranial aneurysm (IA) rupture is a common SAH etiologic factor [4] and IA rupture is among the leading causes of stroke-related morbidity and mortality in the United States [6]. Although mostly asymptomatic, a small percentage of IA can rupture and have devastating health consequences [6]. The main impetus for IA therapies prior to rupture has been to prevent future IA rupture which could trigger SAH and its associated morbidity and mortality risks [4-6]. Two established therapies for patients presenting with an IA include surgical clipping [7] and endovascular coiling [7-8]. Both therapies are aimed at excluding the aneurysm from the intracranial circulation without occluding normal vessels [9]. While surgical clipping is less expensive than endovascular coiling [10-11], endovascular coiling has exhibited a better safety profile compared to surgical clipping [12-13]. However, IA occlusion rate -- a measure of treatment effectiveness -- was shown to be lower among patients treated with endovascular coiling than among those treated with surgical clipping, potentially increasing the need for retreatment via recanalization [14-15]. In recent years, different types of endovascular treatments became available, including stent- and balloon-assisted coiling (SAC and BAC) [16-17]. In rare instances, combined treatments occur, especially when patients who fail endovascular therapy are treated with surgical clipping [9, 18].

Previously conducted clinical trials and observational studies have mainly examined risks of various complications, including ischemic stroke, in the context of surgical clipping or endovascular coiling. By contrast, research has been more limited in the context of evaluating ischemic stroke rates among patients undergoing endovascular coiling with or without adjunctive techniques such as BAC or SAC or combined treatments using surgical clipping with endovascular coiling [19-20]. The overall risk of ischemic stroke after surgical and endovascular repair of unruptured IA remains uncertain. Current evidence suggests that the rate of ischemic stroke may differ according to clinical presentation and treatment selection. Ischemic events can result from detachment of emboli from the IA wall, manifesting as transient ischemic attacks and ischemic events from distal embolization is one of the indications for IA treatment [17]. Furthermore, comorbid conditions that are prevalent in elderly patients, including ischemic stroke, may be a contraindication for surgical clipping, whereas endovascular coiling utilizes thrombogenic coils potentially increasing the risk of ischemic stroke [17]. 

Ischemic stroke among hospitalized patients who underwent surgical or endovascular therapies for unruptured IA (surgical clipping, endovascular coiling, SAC or BAC, combined surgical clipping, and endovascular coiling) has not been previously examined in nationally representative samples. The purpose of this cross-sectional study is to compare perioperative ischemic stroke rates and in-hospital outcomes among hospitalized patients with unruptured IA according to treatment selection using data from the 2002-2012 Nationwide Inpatient Sample (NIS). We hypothesized that the rate of ischemic stroke varies according to the type of treatment selection, independent of patient- and hospital-level characteristics. We also hypothesized that an ischemic stroke diagnosis may modify the relationship between treatment selection and in-hospital outcomes.

## Materials and methods

Secondary analyses were performed using the Agency for Healthcare Research and Quality (AHRQ) Healthcare Cost and Utilization Project (HCUP) NIS, the largest public-use all-payer database in the United States that provides national estimates for healthcare-related outcomes. The NIS database contains data elements typical of a discharge abstract. It was originally generated through 20% stratified probability sampling of community hospitals from the AHRQ HCUP State Inpatient Databases, whereby all hospital discharge records were kept. Since 2012, the 20% NIS sampling strategy shifted to hospital discharge records from HCUP-participating hospitals. The NIS sampling frame was divided into strata based on the following hospital characteristics: ownership/control, bed size, teaching status, urban/rural location, and U.S. region. Subsequently, sampling was performed with probabilities proportionate to the number of hospitals (or hospital discharges) in each stratum. The AHRQ HCUP was approved by an Institutional Review Board in accordance with the Declaration of Helsinki.

Hospital discharge records were selected from the 2002-2012 NIS and specific inclusion and exclusion criteria were applied to define the study population. Inclusion criteria were defined as follows: (1) underwent surgical or endovascular treatment for intracranial aneurysms (IAs), with at least one of the following ICD-9-CM procedure codes: (a) surgical clipping [“clipping of aneurysm” (39.51)]; (b) endovascular coiling {“other repair of aneurysm” [(39.52) or (‘88.41’ and no ‘39.51’)]; “endovascular repair or occlusion of head and neck vessels” (39.72); “other endovascular repair (of aneurysm) of other vessels” (39.79); “endovascular embolization or occlusion of vessel(s) of head or neck using bare coils” (39.75); “endovascular embolization or occlusion of vessel(s) of head or neck using bioactive coils” (39.76)}; (c) SAC (intracranial stenting (00.65) with coiling (39.52, 39.79 or 39.72) of cerebral aneurysms); (d) BAC (partial occlusion (balloon) (temporary) (39.77) with coiling (39.52, 39.79, or 39.72) of cerebral aneurysms) and (2) was diagnosed with unruptured IAs [unruptured intracerebral aneurysm (437.3)]. Furthermore, hospital discharge records were excluded if at least one of the following ICD-9-CM diagnostic codes was identified: (a) ruptured syphilitic aneurysm (094.87); (b) cerebral arteritis (437.4); (c) arteriovenous malformation or fistula (747.81); (d) traumatic hemorrhage (800.0-801.9, 803.0-804.9, 850.0-854.1, 873.0-873.9); (e) treatment diagnosis for arteriovenous malformation repair or radiosurgery [39.53 or 92.30 (923.x)]. Case definition was based on published literature that examined IA treatment outcomes using national databases [21]. It is worth noting that flow diverters (e.g. Pipeline Embolization Device) were not considered in these analyses as they became available towards the end of the study period of interest.

 Using ICD-9-CM procedure codes, we categorized the exposure variable (treatment selection) among hospital discharge records into four groups: (1) surgical clipping alone (referent); (2) endovascular coiling alone; (3) SAC or BAC; (4) surgical clipping and endovascular coiling. The outcome variable of interest was defined as hospital discharge records having at least one of the following ICD-9-CM diagnosis of ischemic stroke: 434.91 (cerebral artery occlusion, unspecified with cerebral infarction); 434.11 (cerebral embolism with cerebral infarction); or 434.01 (cerebral thrombosis with cerebral infarction). In-hospital outcomes were defined as nonroutine discharge (defined using the “dispuniform” variable) and loge-transformed length of hospital stay (in days). A priori confounders of the hypothesized relationship between the exposure and outcome variables were categorized as patient- and hospital-level characteristics. Patient-level confounders included sex, age, race/ethnicity, and IA rupture status, Elixhauser comorbidity score, household income, obesity diagnosis, elective admission status, year of admission, admission quarter, weekend admission status, and primary payer. Hospital-level confounders were defined as hospital region, hospital control, teaching status, and hospital bed size.

The Statistical Analysis System (SAS) version 9.4 (SAS Institute, Cary, NC) and STATA version 15 (StataCorp, College Station, TX) were used to analyze the NIS data, considering the complex survey design. First, we estimated ischemic stroke rate according to the patient- and hospital-level characteristics. Second, we evaluated the association between treatment selection and ischemic stroke, before and after controlling for a priori confounders. Third, we examined the joint effects of treatment selection and ischemic stroke diagnosis on in-hospital death and length of hospital stay, after controlling for patient- and hospital-level characteristics. Descriptive statistics included mean (± standard error) for continuous variables and percentages for categorical variables. Bivariate relationships were evaluated using uncorrected Chi-square and design-based F-tests, as appropriate. Linear and logistic regression models were constructed to estimate crude and adjusted β coefficients, odds ratios (cORs and aORs) with their 95% confidence intervals (CIs). Two-sided statistical tests were conducted, whereby p < 0.05 was considered statistically significant.

## Results

**Figure 1:**
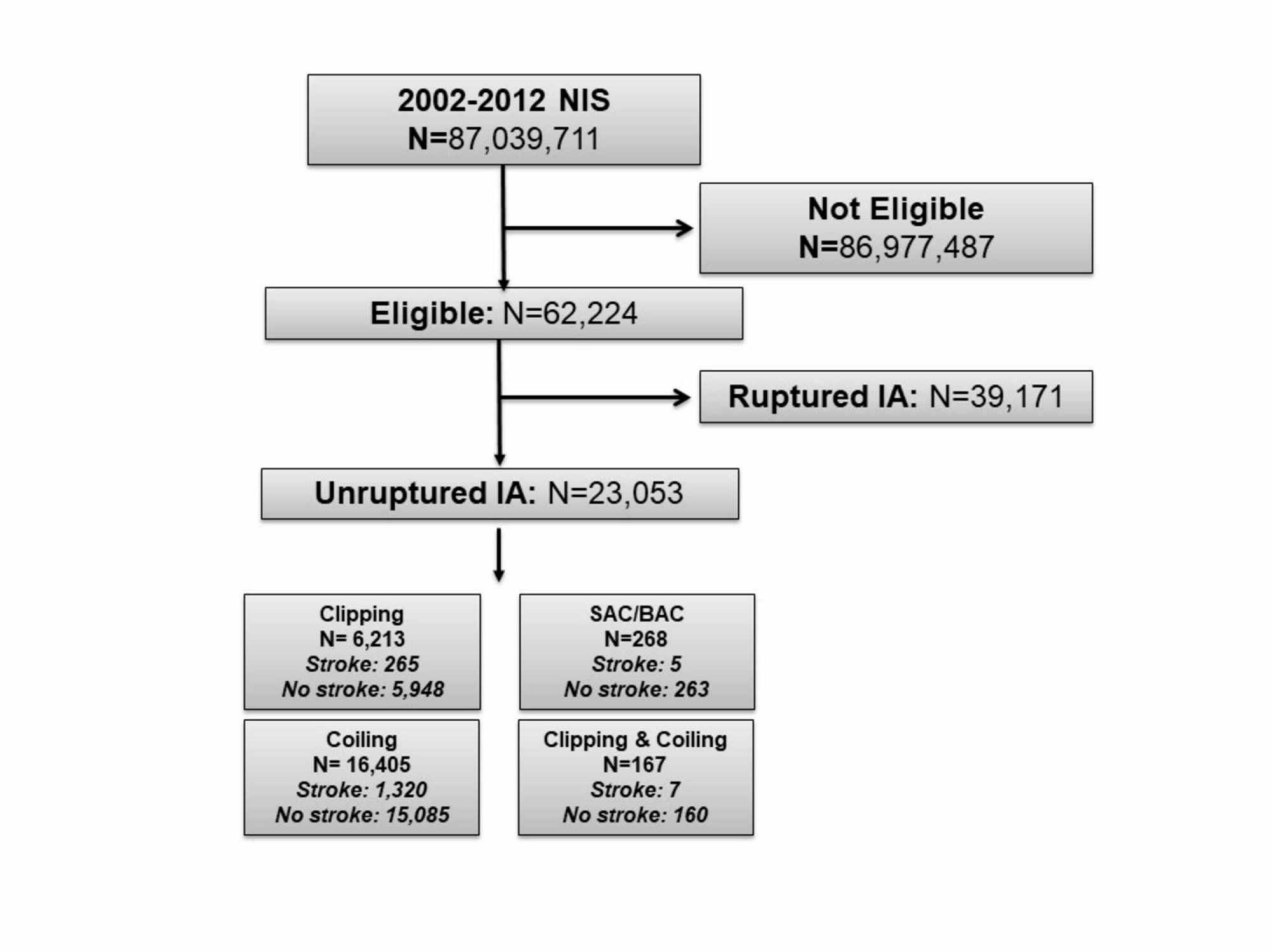
Study flowchart – 2002-2012 NIS. NIS, Nationwide Inpatient Sample; BAC, balloon assisted coiling; SAC, stent assisted coiling; IA, intracranial aneurysm.

The 2002-2012 NIS database included 87,039,711 hospital discharges, of which 23,053 met all eligibility criteria. A total of 1,597 study-eligible hospital discharges had ischemic stroke as one of the diagnoses. Furthermore, 6,213 underwent surgical clipping, 16,405 endovascular coiling, 268 BAC or SAC, and 167 combined treatment with surgical clipping and endovascular coiling (Figure 1).

The majority of the study population, with a mean age of 57 years, consisted of female (74%), White (74%), nonobese (96%) patients who had no co-morbidities (99.9%), were admitted nonelectively (65%), on weekdays (93%), to Southern (39%), teaching hospitals (85%) with a large number of beds (83%). Primary payers were frequently reported as private insurance (46%) or Medicare (34%). The overall rate of ischemic stroke was 6.9% with variations observed according to classes of patient and hospital characteristics. On average, patients diagnosed with ischemic stroke were significantly older than those who were not diagnosed with ischemic stroke. Also, ischemic stroke rate was higher in males, those having Black race/ethnicity, or an Elixhauser Comorbidity Score of 1 or more. Nonelective admission, weekend admission, and private not-for-profit hospital control were also associated with increased ischemic stroke rate. Ischemic stroke rate varied significantly over the years, increasing from 5.3% in 2002 to 11.9% in 2012, with seasonal variations based on admission quarter (Table 1).

**Table 1:** Perioperative ischemic stroke according to patient- and hospital-level characteristics among patients with unruptured IAs (n=23,053). SEM, standard error of the mean; IAs, intracranial aneurysms

	Ischemic stroke		
	%	% Yes	aOR (95% CI)
Overall:	100	6.9	
Sex:	<0.0001		
Male	26.3	8.5	Ref.
Female	73.7	6.4	0.77 (0.69, 0.87)
Age (years): mean ± SEM	<0.0001		
Overall:	57.2 ± 0.09		1.03 (1.02, 1.04)
Stroke	63.2 ± 0.35		
No stroke	56.8 ± 0.09		
Race/Ethnicity:	<0.0001		
White	72.9	6.4	0.71 (0.61, 0.83)
Black	12.1	9.5	Ref.
Hispanic	9.2	7.1	0.70 (0.56, 0.88)
Other	5.7	7.9	0.80 (0.62, 1.03)
Elixhauser Comorbidity Score:	<0.0001		
0	99.9	6.9	Ref.
1+	0.07	37.7	3.92 (1.16, 13.27)

Household income (quartile):	0.58		
1^st^	23.7	7.4	Ref.
2^nd^	23.8	6.9	1.00 (0.86, 1.17)
3^rd^	24.1	6.8	0.95 (0.82, 1.12)
4^th^	21.9	6.9	0.97 (0.83, 1.15)
Unknown	6.5	6.4	1.03 (0.72, 1.46)
Obesity diagnosis:	0.20		
Yes	4.5	7.9	1.27 (0.99, 1.63)
No	95.5	6.9	Ref.
Elective admission status:	<0.0001		
Nonelective	64.9	14.5	Ref.
Elective	35.0	2.9	0.21 (0.19, 0.24)
Admission year:	<0.0001		
2002	4.2	5.7	Ref.
2003	7.5	4.0	0.92 (0.55, 1.52)
2004	6.7	5.3	1.23 (0.75, 2.03)
2005	7.1	6.0	1.17 (0.72, 1.91)
2006	6.9	6.9	1.42 (0.88, 2.31)
2007	6.4	7.3	1.38 (0.85, 2.25)
2008	11.5	7.8	1.66 (1.05, 2.65)
2009	9.7	8.6	1.84 (1.15, 2.94)
2010	13.4	8.3	1.94 (1.22, 3.06)
2011	13.3	6.9	1.55 (0.98, 2.48)
2012	13.3	7.1	1.69 (1.06, 2.70)
Admission quarter:	0.22		
1^st^ quarter	25.0	6.5	Ref.
2^nd^ quarter	25.3	7.3	1.18 (1.02, 1.38)
3^rd^ quarter	25.1	7.4	1.16 (0.99, 1.35)
4^th^ quarter	24.6	6.7	1.08 (0.93, 1.26)
Weekend admission status:	<0.0001		
Monday-Friday	92.8	6.1	Ref.
Saturday-Sunday	7.1	18.0	1.44 (1.24, 1.68)
Primary payer:	<0.0001		
Medicare	34.1	9.5	Ref.
Medicaid	11.0	6.8	1.14 (0.92, 1.40)
Private insurance	45.8	4.9	0.99 (0.86, 1.16)
Self-pay	3.7	8.9	1.25 (0.95, 1.65)
No charge	0.8	7.8	1.15 (0.64, 2.05)
Other	3.7	7.1	1.27 (0.94, 1.70)
Hospital region:	0.89		
Northeast	25.2	7.0	Ref.
Midwest	16.7	7.2	1.18 (0.99, 1.39)
South	38.8	6.8	0.95 (0.82, 1.09)
West	19.2	7.1	1.09 (0.93, 1.30)
Hospital control:	<0.0001		
Government or private	76.8	6.5	Ref.
Government, nonfederal	1.3	9.4	1.47 (0.92, 2.37)
Private, not-for-profit	6.1	10.3	1.48 (1.09, 2.01)
Private, investor-owned	2.2	11.7	1.32 (0.91, 1.93)
Private	0.2	2.1	0.28 (0.04, 2.29)
Unknown	13.3	7.1	--
Teaching status:	<0.0001		
Rural	1.4	8.0	Ref.
Urban – nonteaching	13.3	10.2	0.84 (0.52, 1.36)
Urban – teaching	85.3	6.4	0.87 (0.54, 1.39)
Hospital bed size:	0.10		
Small	3.6	6.8	Ref.
Medium	13.8	7.9	0.93 (0.67, 1.2)
Large	82.6	6.8	0.85 (0.63, 1.15)

Table 2 displays treatment selection and its relationship to rate of perioperative ischemic stroke, before and after controlling for confounders. The rate of ischemic stroke varied significantly (p < 0.0001) according to treatment selection, as follows: surgical clipping (4.3%), endovascular coiling (8.1%), BAC or SAC (1.9%), and combination of surgical clipping with endovascular coiling (4.2%). Compared to surgical clipping, endovascular coiling was associated with higher rate of ischemic stroke before (cOR=1.96, 95% CI: 1.72, 2.27), but not after (aOR=0.97, 95% CI: 0.84, 1.12) controlling for confounders. Multivariable logistic regression models suggested that unruptured IA patients who underwent SAC or BAC were less likely to have experienced ischemic stroke compared to surgical clipping alone (aOR=0.34, 95% CI: 0.14, 0.85). By contrast, patients who underwent combined treatment with surgical clipping and endovascular coiling had similar rate of ischemic stroke when compared to those who underwent surgical clipping alone.

**Table 2:** Treatment selection and its relationship to perioperative ischemic stroke among patients with unruptured aneurysms (n=23,053). OR, odds ratio; CI, confidence interval; SAC, stent assisted coiling; BAC, balloon assisted coiling. ^a^Adjusted for patient-level and hospital-level characteristics.

	% Ischemic stroke	Unadjusted		Adjusted^a^	
	p < 0.0001	OR	95% CI	OR	95% CI
Surgical clipping	4.3	Ref.	--	--	--
Endovascular coiling	8.1	1.96	1.72, 2.27	0.97	0.84, 1.12
SAC or BAC	1.9	0.43	0.18, 1.06	0.34	0.14, 0.85
Clipping and coiling	4.2	0.97	0.45, 2.08	0.83	0.37, 1.83

Table 3 displays a stratified analysis of treatment selection as predictor of in-hospital outcomes, namely nonroutine discharge and loge-transformed length of hospital stay according to ischemic stroke diagnosis. Compared to surgical clipping, endovascular coiling as well as SAC and BAC recipients were less likely to experience nonroutine hospital discharge, regardless of ischemic stroke diagnosis. Furthermore, loge-transformed length of hospital stay was shorter among recipients of endovascular coiling as well as SAC and BAC and longer among recipients of combined treatments as compared to those who underwent surgical clipping. There was a significant interaction between treatment selection and ischemic stroke diagnosis, when endovascular coiling was compared to surgical clipping, but not for SAC and BAC or combined treatments.

 

**Table 3:** Treatment selection and its relationship to nonroutine discharge and length of hospital stay according to ischemic stroke status among patients with unruptured aneurysms (n=23,053). OR, odds ratio; CI, confidence interval; SAC, stent assisted coiling; BAC, balloon assisted coiling. Interaction effect between stroke and treatment selection: nonroutine disposition (stroke x endovascular coiling: p < 0.0001; stroke x SAC or BAC: p = 0.87; stroke x  clipping and coiling: p = 0.98); length of hospital stay (stroke x endovascular coiling: p < 0.0001; stroke x SAC or BAC: p = 0.86; stroke x  clipping and coiling: p = 0.36). ^a^Adjusted for patient-level and hospital-level characteristics.

	Nonroutine discharge^a^		Log_e _length of hospital stay^a^	
	Total			
	OR	95% CI	β	95% CI
Treatment selection:				
Surgical clipping	Ref.	--	Ref.	--
Endovascular coiling	0.31	0.29, 0.34	-0.91	-0.93, -0.89
SAC or BAC	0.15	0.09, 0.24	-0.89	-0.97, -0.79
Clipping and coiling	1.35	0.94, 1.93	0.11	0.0065, 0.21
	Stroke			
	OR	95% CI	β	95% CI
Treatment selection:				
Surgical clipping	Ref.	--	Ref.	--
Endovascular coiling	0.27	0.19, 0.39	-0.78	-0.88, -0.67
SAC or BAC	0.13	0.021, 0.85	-0.87	-1.61, -0.13
Clipping and coiling	1.58	0.21, 11.82	0.45	0.067, 0.85

	No stroke			
	OR	95% CI	β	95% CI
Treatment selection:				
Surgical clipping	Ref.	--	Ref.	--
Endovascular coiling	0.29	0.27, 0.33	-0.90	-0.92, -0.88
SAC or BAC	0.16	0.092, 0.26	-0.86	-0.95, -0.77
Clipping and coiling	1.39	0.96, 2.02	0.10	0.0048, 0.19

## Discussion

In this cross-sectional study using a nationally representative sample of hospital discharge records, we estimated the rate and in-hospital outcomes of perioperative ischemic stroke according to treatment selection among hospitalized patients with unruptured IA. Our results suggested that the overall rate of ischemic stroke was approximately 7% ranging between 2% for SAC or BAC and 8% for endovascular coiling alone. Ischemic stroke rate in the context of coiling appears to be high. However, it is likely that patients treated with coiling were older and may have had cerebral infarcts prior to coiling. It is also likely that patients with cerebral infarcts could only be treated with coiling taking into consideration the risk-benefit ratio among older populations. Compared to surgical clipping, ischemic stroke appears to be less commonly reported among patients who underwent SAC or BAC, after controlling for patient and hospital-level characteristics. Lower cerebral infarct rates in SAC or BAC were likely due to more experienced endovascular radiologists performing these expensive treatments and the possibility that patients were specifically selected for these treatments. As expected, our results also suggested that, regardless of ischemic stroke diagnosis, nonroutine hospital discharge was less frequent and hospital stay was shorter among patients who underwent endovascular coiling as well as those who underwent SAC or BAC, when compared to those who underwent surgical clipping. Patients who underwent combined treatments had, as expected, longer hospitalizations as compared to those who underwent surgical clipping.

Because diagnostic data were obtained from hospital discharge records, ischemic stroke rate in this population of patients undergoing surgical or endovascular therapies for unruptured IA may have been underestimated. Although all ischemic stroke events should be recorded in the context of invasive treatments for scientific and clinical purposes, it is likely that many of the reported cases were symptomatic, and that minor or asymptomatic ischemic stroke events were missed in the absence of post-treatment imaging. In fact, healthcare professionals with differing levels of expertise and potential for conflict of interest have completed these hospital discharge records. The absence of post-treatment imaging may be attributed to the desire of treating physicians not to report cerebral infarcts unless the patient clearly shows symptoms. Furthermore, differences in ischemic stroke rates among treatment groups may be attributed to uncontrolled confounding by baseline characteristics that could not be ascertained through the NIS database. According to a prospective multicenter International Study of Unruptured Intracranial Aneurysms study involving 1,692 patients who did not have aneurysmal repair, 1,917 patients who had surgical clipping, and 451 who had endovascular coiling, post-treatment IA rupture was associated with age (among clipping cases only), IA treatment history, IA size, and location (among clipping and coiling cases) [22].

 Unlike previously conducted studies, we found that the rate of ischemic stroke did not differ between the major treatment methods, namely surgical clipping and endovascular coiling, after controlling for confounders. By contrast, the finding that surgical clipping had worse in-hospital outcomes than endovascular coiling with significant interaction by ischemic stroke diagnosis is consistent with existing studies. Comparative studies suggest that clinical outcomes are generally better among patients treated with endovascular coiling versus surgical clipping [1, 23]. For instance, a greater likelihood of experiencing ischemic and hemorrhagic complications among patients treated with clipping compared to those treated with coiling was previously reported in a large retrospective study involving 4,899 unruptured IA patients (1388 clipping, 3551 coiling) selected from 120 hospitals using the Premier Perspective database [24]. A previously published systematic review of 24 studies focused on unruptured IA found that coiling was superior to clipping in terms of short-term disability, neurological and cardiovascular complications [7]. Nearly three-quarters of study-eligible patients identified in our study had undergone endovascular coiling. This trend is in line with better clinical outcomes among patients receiving this therapy, despite relatively high ischemic stroke rate [25].

Similarly, we found that patients undergoing combined therapies and those undergoing surgical clipping for unruptured IA did not experience different rates of ischemic stroke. Previous research indicated that combined surgical and endovascular treatments may be safe and effective [18]. In a cohort study involving 111 patients who underwent clipping of previously coiled IA, 2.7% had major complications and underwent coil extraction as a result of post-operative stroke [26]. By contrast, we found that ischemic stroke was less frequent in the context of SAC or BAC as compared to surgical clipping alone among patients with unruptured IA. This finding is contrary to a previously conducted systematic review and meta-analysis of 114 studies (106,433 patients with 108,263 aneurysms), whereby the use of stenting or SAC in the context of endovascular therapies increased the complication rates by nearly two to three-fold [27]. In a previously conducted study, McDonald et al. performed secondary analyses of the 2004-2008 NIS database to compare in-hospital outcomes of endovascular coiling with and without adjunctive stenting among patients with unruptured IA [28]. Although their study did not assess ischemic stroke, per se, the two treatment groups experienced similar hospitalization outcomes, including rates of in-hospital death and discharge to a care facility [28].

 Our study findings should be interpreted with caution and in light of several limitations. First, the NIS administrative database consists of hospital discharge records, which are often limited in scope, completeness, and accuracy. This database was not designed specifically to address the research question under study and may be more prone to biases than prospective cohort studies involving patient samples. For instance, ischemic stroke is self-reported by institutions in the NIS and is prone to error in the absence of tight scrutiny by a clinical events committee or data safety and monitoring board. Center-specific diagnosis and treatment algorithms may cause variability in the sensitivity of diagnosing symptomatic or even non-symptomatic strokes depending on imaging modalities used. Also, detailed information is lacking concerning IA location, pre-treatment condition of patients, stroke etiology and other factors that may affect the interpretation of treatment-outcome relationships. Second, data clustering as a result of patient re-admission to one of the NIS-participating hospitals cannot be evaluated in the absence of unique patient identifiers. Third, complete subject analysis was performed with potential for selection bias because of missing exposure, outcome and/or covariate data. Fourth, eligibility criteria and many of the study variables such as treatment selection, ischemic stroke and obesity diagnosis were defined using previously reported ICD-9-CM codes [21], potentially leading to misclassification bias. Fifth, adjusted measures of association may be biased due to unmeasured confounding in the context of an observational design. Key confounders including IA size, location and morphology, are often collected in clinical trials but cannot be ascertained using ICD-9-CM codes from hospital discharge records. Although pre-treatment condition cannot be ascertained using NIS data, it is plausible that patients who underwent two therapies or adjunctive treatments may have worse prognosis than those who underwent one type of therapy. Fifth, analyses were based on relatively old (2002-2012) NIS data, and could not capture users of more advanced endovascular techniques such as flow diverters. Finally, the cross-sectional design cannot establish a temporal or causal relationship between the primary exposure (treatment selection) and outcome (ischemic stroke) variables of interest. Moreover, the duration of time between onset of IA symptoms and treatment is unknown and the follow-up duration was limited by the length of hospital stay. As the purpose of the study was to evaluate the rate and in-hospital outcomes of ischemic stroke among hospitalized patients according to treatment selection, this may not be a major issue. Despite these limitations, study findings are not likely due to chance given the large number of hospital discharge records available in the NIS database.

## Conclusions

In conclusion, no differences in ischemic stroke rates were observed for the two major therapies for unruptured IA, namely endovascular coiling and surgical clipping. The rate of perioperative ischemic stroke among patients diagnosed with unruptured IA appears to be less for SAC or BAC versus surgical clipping. Ischemic stroke diagnosis influenced the magnitude of the association between treatment selection and in-hospital outcomes, namely nonroutine discharge and length of stay, only when the two main therapies were compared. Further research is needed to distinguish pre-existing, concomitant, and post-operative ischemic stroke using a prospective cohort design, and taking into consideration IA size, location, and morphology unruptured IA patients. 
